# Structure and Infrared Emissivity Properties of the MAO Coatings Formed on TC4 Alloys in K_2_ZrF_6_-Based Solution

**DOI:** 10.3390/ma11020254

**Published:** 2018-02-07

**Authors:** Guangrui Gao, Ying Li, Dan Hu, Zhengping Xi

**Affiliations:** 1 School of Metallurgy, Northeastern University, Shenyang 110819, China; mayanyan0814@163.com; 2 Northwest Institute for Nonferrous Metal Research, Xi’an 710016, China; 3 Xi’an Surface Material Protection Co, Ltd., Xi’an 710016, China; xiansfs@126.com

**Keywords:** micro-arc oxidation, K_2_ZrF_6_, coating, infrared emissivity

## Abstract

Micro-arc oxidation (MAO) ceramic coatings were formed on TC4 alloy surface in silicate and metaphosphate electrolytes based with K_2_ZrF_6_ for various concentrations. X-ray diffraction (XRD), Scanning electron microscopy (SEM), X-ray photoelectron spectroscopy (XPS) were used to characterize the phase composition, microstructure and chemical compositions of the coatings. The infrared emissivity of the coatings was measured at 50 °C in a wavelength range of 8–20 µm. The microstructural observations all revealed the typical porousstructures. Moreover, adecline in roughness and thickness of the prepared coatings can be observed when the concentration of K_2_ZrF_6_ increases. Combined with the results of XRD and XPS, it was found that all the oxides existed as the amorphous form in the coatings except the TiO_2_ phase. The coatings exhibited the highest infrared emissivity value (about 0.89) when the concentration of K_2_ZrF_6_ was 6 g/L, which was possibly attributed to the defect microstructure and the optimal role of ZrO_2_.

## 1. Introduction

With the development of infrared technology in recent years, the research of high emissivity coatings has become a focus around the world. These coatings have been widely used in various fields such as thermal protection systems and energy-saving in industrial furnace [1,2,3,4,5]. Substances such as carbides (mainly SiC), transition metal oxides (MnO_2_, Fe_2_O_3_, ZrO_2_, Cr_2_O_3_, NiO, TiO_2_, Co_3_O_4_, et al.), as well as ceramics and glass are usually used to prepare high emissivity coatings due to their high infrared emissivity [6,7]. For instance, Li et al. prepared a kind of high infrared emissivity coating by applying the mixture of ZrSiO_4_ and aluminosilicate glass powder to the SiC matrix, and then treated by high temperature sintering. The average emissivity of the coatings in the waveband of 1–22 μm demonstrated the highest value around of 0.93. Furthermore the coatings also had high temperature oxidation resistance and compactness [8]. Likewise, Tang et al. added FeSO_4_ into the electrolyte to prepare the micro-arc oxidation (MAO) coatings with high emissivity on TC4 alloys. The emissivity of the coating with 3 g/L FeSO_4_ reached the maximum 0.87, and its bonding strength was higher than 33 MPa [9].

Up to now, many surface treatment techniques have been adapted to the preparation of high emissivity coatings including brushing, sintering, electro deposition, electroless deposition and sol-gel technique, etc. However, some widespread problems exist such as poor uniformity, low bonding strength and high preparation costs. Micro arc oxidation, also named as Plasma electrolytic oxidation or Micro plasma oxidation, is acknowledged as an advanced surface treatment technology of placing the valve metals or their alloys in electrolytes under the additional high voltage. During the reaction process, the oxide film on the surface is instantly broken down and the appearance of the sparks leads to the formation of the discharge channel, which makes the ceramic coatings grow in situ through the reaction of the electrolytes and the metal substrate. Compared with the traditional anodization, the MAO technology transforms the working area into a high-pressure discharge zone so as to prepare the ceramic coatings, which could simultaneously achieve high wear resistance, better corrosion resistance and stronger binding strength between the coatings and substrate [10,11]. Furthermore, this technology also has the advantages of high production efficiency and less environmental pollution. In addition, the MAO coatings of different composition can be obtained by adjusting the electrolyte composition and process parameters. Therefore, using the MAO technique to deposit the functional coatings has received much attention in recent years [12,13,14].

Ingeneral, the infrared emissivity of MAO coatings is related to the chemical composition, surface condition, coating thickness, and electrolytic parameters, but the most influential factor is the composition. It is worth noting that zirconium oxide has high thermal stability, oxidation resistance and low specificheat as well as thermal conductivity as a kind of transition metal oxide. Several authors have researched the effect of K_2_ZrF_6_ on properties of the ceramic coatings. Zhang et al. observed the MAO coatings fabricated on an aluminum alloy which were produced in the K_2_ZrF_6_-based electrolyte [15]. The study found that the deposition rate of the coatings and their uniformity all increased obviously by adding K_2_ZrF_6_, and the coatings also exhibited the excellent heat resistance. It is further reportedby various researchers that K_2_ZrF_6_ could increase the tensile strength and shear strength of the MAO coatings [16,17]. However, most researches mainly focus on the significant improvement in mechanical properties, thermal shock resistance as well as corrosion resistance of the coatings, and there are few reports about the infrared emission performance [18,19,20]. In this work, the micro-arc oxidation method was carried out on TC4 alloy substrate under the different concentration of K_2_ZrF_6_, the effect of the K_2_ZrF_6_ additives on the phase composition, microstructure, chemical composition and infrared emissivity was systematically investigated. Meanwhile, this paper also includes a further expatiation about the reaction details.

## 2. Materials and Methods

TC4 alloy (Fe ≤ 0.30, C ≤ 0.10, N ≤ 0.05, H ≤ 0.015, O ≤ 0.20, Al: 5.5–6.8, V: 3.5–4.5, all in wt %) was cut into the regular specimens with dimensions of 30 mm × 30 mm × 2 mm and used as the substrate to be treated. These specimens were ground successively to 1500 grit SiC papers, then ultrasonically rinsed with ethyl alcohol and dried in room temperature. The specimens were used as anode and a electrolyser which made of stainless steel served as cathode. In this work, the MAO process was carried out for 40 min using a 300 kW positive pulsed device and the main pulse parameters were fixed as follows: frequency 600 Hz, voltage 500 V, and a duty cycle of 15%. The reaction temperature was cooled by a cooling water system to control the value between 25 °C–40 °C. The electrolytes were composed of Na_2_SiO_3_ (10 g/L), (NaPO_3_)_6_ (6 g/L), NaOH (0.8 g/L), with K_2_ZrF_6_ 0 g/L, 3 g/L, 6 g/L, 9 g/L respectively. After the preparation process, all samples were rinsed with distilled water and dried with the blower.

Phase composition of the coatings was analyzed by a RICOH/max-rB automatic X-ray diffractometer (XRD, D/max-2200pc, RIGAKU, Tokyo, Japan) using a Cu Kα source. The surface and cross-section morphologies of the MAO treated coatings were observed by scanning electron microscopy (SEM, JSM6460, JEOL, Tokyo, Japan), and the chemical compositions of the coatings were measured by energy dispersive X-ray spectrometer (EDS) attached to SEM. X-ray photoelectron spectroscopy (XPS, ESCALAB 250Xi, Thermo Fisher Scientific, Waltham, MA, USA) with an Al Kα anode was utilized to determine the chemical states of the elements. The thickness and roughness of the coatings were respectively measured by eddy current thickness meter (TT 260, Time Company, Beijing, China) and surface roughness tester (TR-200, Time Company, Beijing, China). Fourier transform infrared (FT/IR-6100, JASCO, Tokyo, Japan) was used to measure the infrared emissivity of the coatings at 50 °C.

## 3. Results

### 3.1. Surface and Cross-Section Microstructure

**Figure 1:**
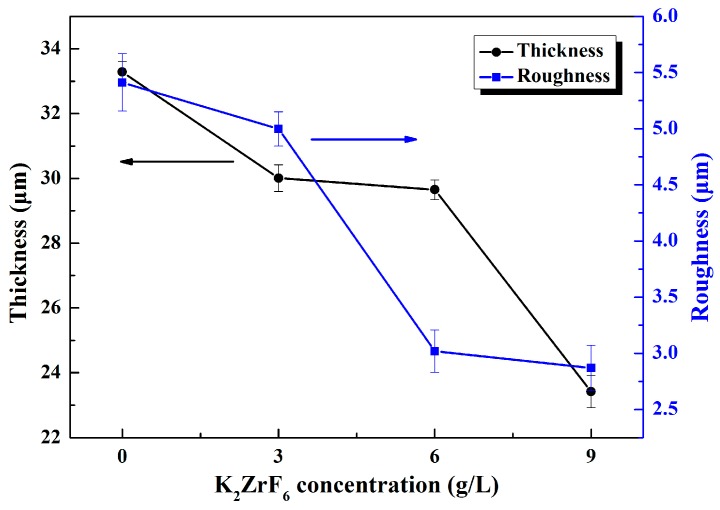
Thickness and roughness of the micro-arc oxidation (MAO) coatings under different K_2_ZrF_6_ concentrations.

Figure 1 shows the thickness and roughness of the MAO coatings formed in different electrolytes. Three samples were tested in each group, 10 points were selected on each sample and the average was taken as the final measurement data. It can be seen that lower thickness and roughness were obtained with the introduction of K_2_ZrF_6_. Furthermore, it can be noted that the thickness did not decline significantly except when the additive quantity of K_2_ZrF_6_ reached 9 g/L and the roughness markedly dropped when the K_2_ZrF_6_ concentration is up to 6 g/L. The reason for this may be attributed to the inhibition of K_2_ZrF_6_ on the deposition of silicate and metaphosphate on the surface of the coatings.

**Figure 2:**
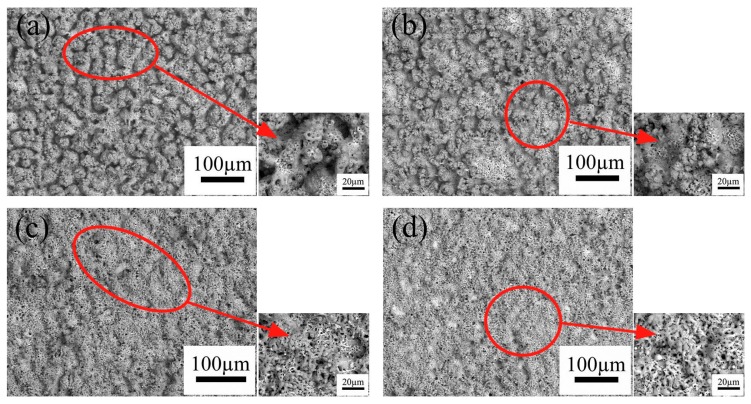
Surface morphologies of various MAO coatings formed in different K_2_ZrF_6_ concentrations: (**a**) 0 g/L; (**b**) 3 g/L; (**c**) 6 g/L; (**d**) 9 g/L.

Figure 2 displays the surface morphologies of the MAO coatings prepared under various conditions. All the coatings possess a porous structure with many crater-shaped micro-pores irregularly distributed and the appearance of this structure is due to the molten oxides and the gas bubbles of the discharge phenomena in the processes. In addition, the surface morphologies as shown in Figure 2 exhibit an obvious difference. With an increase in K_2_ZrF_6_ concentration, the roughness of the coatings decreases, which is consistent with the results of Figure 1. From Figure 2a, large spherical particles with the diameter of several micrometers distribute on the surface. As the concentration of K_2_ZrF_6_ increase to 3 g/L, some protrusions have a tendency to become smooth as shown in Figure 2b. A clearly transformation occurs when the concentration of K_2_ZrF_6_ reaches 6 g/L and 9 g/L (Figure 2c,d), and the spherical protrusions gradually change towards to the dense skeleton structure and the number of pores apparently increase. This is because of the more intensive discharge reaction which was triggered by the enhancement of the electrical conductivity with K_2_ZrF_6_ addition. All the details of changes can be observed from the enlarged small diagrams more clearly. According to the previous studies, silicate is considered to play the dominant role in the coating growth, and metaphosphate could promote the penetrating discharges so as to improve the compactness of the coatings [21,22]. According to the measurement results of the MAO coatings thickness, it is clear that the K_2_ZrF_6_ additive inhibited the deposition of silicate on the surface of coatings.

**Figure 3:**
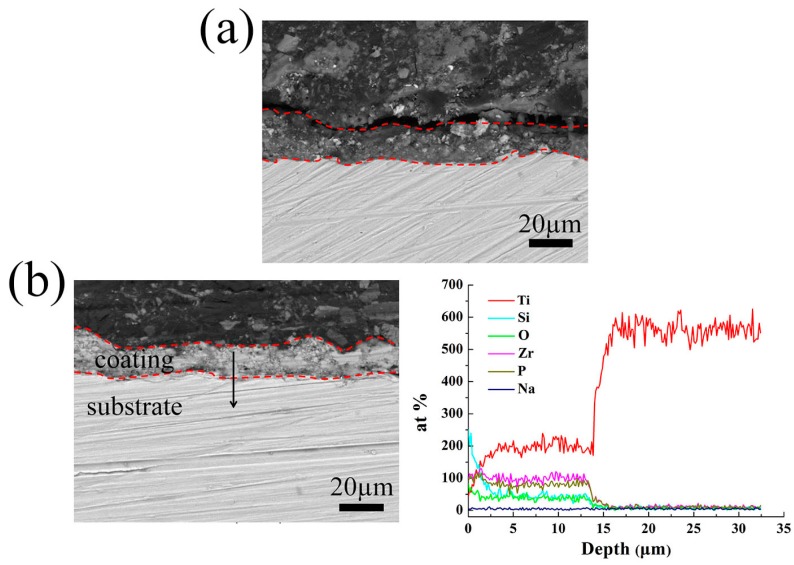
Cross-sectional morphologies and elemental distribution of the MAO coatings formed in different K_2_ZrF_6_ concentrations: (**a**) 0 g/L; (**b**) 9 g/L.

Because the thickness of the coatings changes considerably at the K_2_ZrF_6_ concentration of 9 g/L, here, as shown in Figure 3, the coatings prepared under the K_2_ZrF_6_ addition of 0 g/L and 9 g/L were presented as the examples to explore the cross-sectional morphology and the elemental distribution. The thickness of the coatings decreases obviously with the maximum content of K_2_ZrF_6_ in the electrolyte, but they all have good adhesion with substrate. The coatings all exhibit a relatively dense structure with only a few micro-pores and cracks. In Figure 3b, it can be seen that the main chemical elements of the coating are consistent with the EDS results. Take the binding surface of the coating and substrate as the boundary, the content of Ti increases rapidly, while the opposite trend is observed from elements Si, P, and Zr.

Table 1 shows the elemental distribution developed on the coatings surface of Figure 2. Generally speaking, the type and content of the elements in coatings are closely related to the composition of the electrolyte. It can be seen that the elements O, Si, P, Ti, Zr are the main content compared with other elements in the coatings. When the concentration of K_2_ZrF_6_ increases from 0 g/L to 9 g/L, while the content of Si element decreases quickly from 19.29% to 10.81%. Consequently, it can be concluded that the MAO coatings were mainly formed by the deposition of silicate compounds and the K_2_ZrF_6_ additive evidently inhibited this deposition which provides the basis to explain the changes of the surface morphologies. In contrast, the content of P, Ti, Zr slightly increases. The increase of Zr content is due to the increase of K_2_ZrF_6_ addition in the electrolyte, while the increase content of elements P and Ti is because of the enhancement of the oxidation process.

### 3.2. Phase Composition

**Figure 4:**
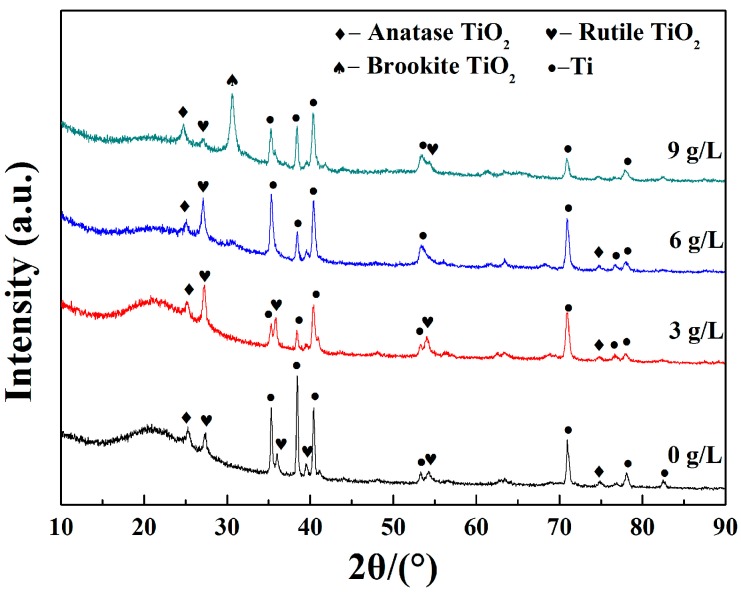
X-ray diffraction (XRD) patterns of MAO coatings formed under various K_2_ZrF_6_ concentrations.

Figure 4 shows the XRD patterns of the prepared coatings at various K_2_ZrF_6_ concentrations. It can be observed that the coatings are composed of anatase-TiO_2_, rutile-TiO_2_, brookite-TiO_2_ and Ti. Many researchers have suggested that the reaction between (OH^−^) ions and (Ti^4+^) ions in the discharge channel could be able to form TiO_2_ phases of different types [9,10,11,12,13,15,16,17,21]. Peaks conformed to Ti substrate appeared because the testing depth was higher than the thickness of the coatings. From the XRD pattern, it can also be seen that when the concentration of K_2_ZrF_6_ reaches 9 g/L, the peak of brookite-TiO_2_ can be detected and the presence is probably due to the temperature changing during the reaction process. Brookite-TiO_2_ has lower reflectivity compared to anatase-TiO_2_ and rutile-TiO_2_, which could make a negative impact on the infrared emissivity of the coatings. In addition, no peaks corresponding to Zr-related, Si-related and P-related species were observed. It is deduced that these groups possibly existed as the amorphous phase. With the increase of K_2_ZrF_6_ addition, all the diffraction peaks become sharper and the radian of the steamed bread peak is gradually weakened, which indicated that the crystallinity of the samples tends to be better.

**Figure 5:**
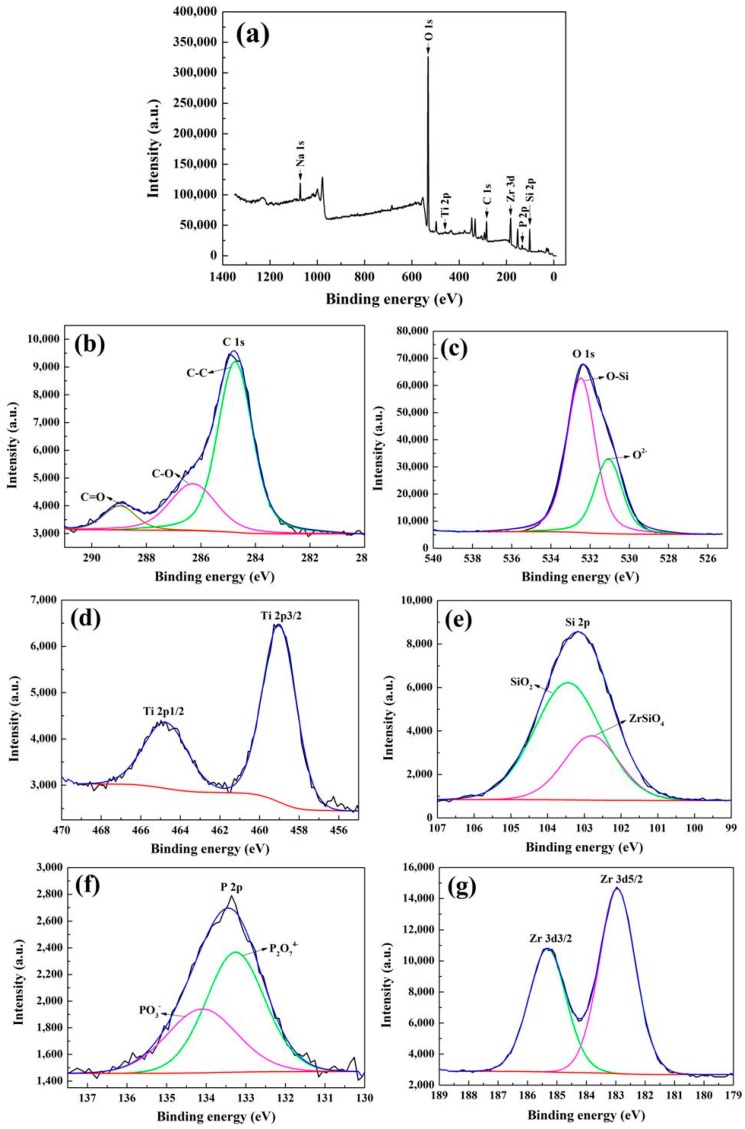
X-ray photoelectron spectroscopy (XPS) spectra of MAO coatings prepared with K_2_ZrF_6_ concentrations of 6 g/L: (**a**) Survey; (**b**) C 1s; (**c**) O 1s; (**d**) Ti 2p; (**e**) Si 2p; (**f**) P 2p; (**g**) Zr 3d.

The XPS measurement was employed to confirm the chemical composition of the MAO coatings. A clean coating surface was obtained by sputtering with Ar ions for 2 min before testing. In addition, the binding energies of all elements were corrected in reference to the C 1s peak at 284.6 eV. The survey spectrum of the coating prepared in the electrolyte of 6 g/L K_2_ZrF_6_ and its high-resolution spectra of major elements are shown in Figure 5. The survey spectrum discloses that the major elements in coatings are O, P, Si, Zr, Ti, Na, which is consistent with the EDS results. The C 1s spectrum shows three peaks C-C, C-O and C=O at the bonding energy 284.6 eV, 286.1 eV and 288.8 eV respectively [23]. The O 1s spectrum can be divided into two peaks at 532.5 eV and 530.8 eV, the first peak is assigned to the Si-O bond of SiO_2_ [24], while the second peak at 530.8 eV relates to O^2−^ [25,26], which corresponds with the oxides in the coatings. Two peaks of Ti 2p spectrum which are located at 464.8 eV and 458.9 eV assert the existence of TiO_2_ [27]. The Si 2p spectrum exhibits two peaks at the bonding energy 102.8 eV and 103.4 eV, the peak at 103.4 eV corresponds to SiO_2_, the second peak at 102.8 eV is attributed to ZrSiO_4_ [28,29], the appearance of ZrSiO_4_ indicates the more complex reactions in the preparation process. P 2p peaks are well fitted at 133.4 and 134.1 eV, which represent the PO_3_^−^ and P_2_O_7_^4−^ separately. The ratio of the area of the two peaks is 50.27:49.73, and therefore indicates that half of the PO_3_^−^ has converted to P_2_O_7_^4−^ [30,31]. The Zr 3d3/2 peak at 185.3 eV and Zr 3d5/2 peak at 182.9 eV are assigned to ZrO_2_ [32]. Combined with the results of EDS, XRD and XPS, the elements Si, P, Zr were all involved in the growth of the coatings and existed in the amorphous state.

### 3.3. Infrared Emissivity Characterization

**Figure 6:**
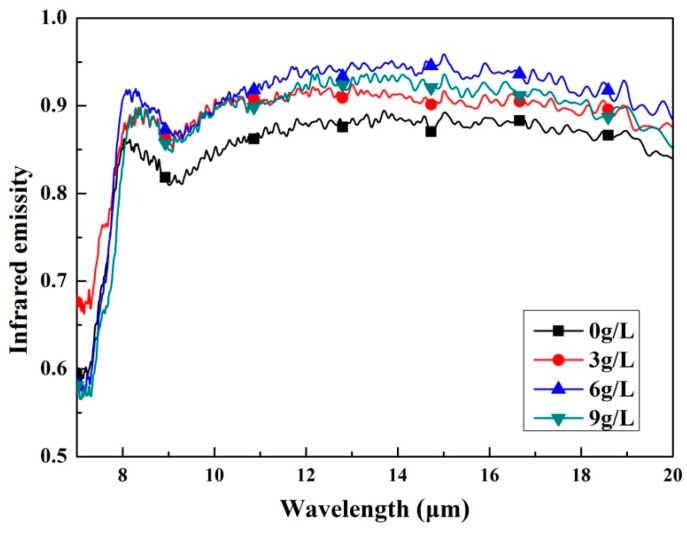
Infrared emissivity curves of the MAO coatings with different K_2_ZrO_4_ concentrations within a waveband of 7–20 μm.

**Figure 7:**
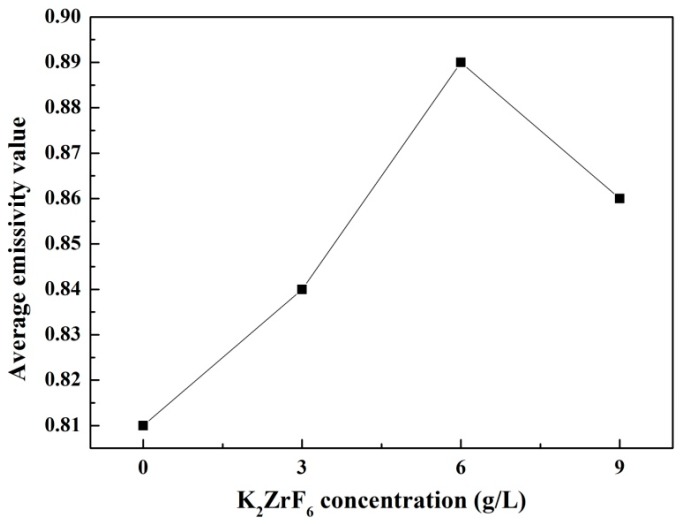
Average infrared emissivity curves of the MAO coatings under different K_2_ZrF_6_ concentrations.

The emissivity is a property which characterizes the radiation properties of the objects, it reveals the radiation radiates by the given body as compared with a blackbody. Higher emissivity is beneficial to the objects for their application in the field of radiative thermal protection. Figure 6 shows infrared emissivity curves measured in the wavelength of 7–20 μm of the as-prepared coatings. According to Figure 6, the coatings formed in the electrolytes added with K_2_ZrF_6_ have obviously higher infrared emissivity compared with the coating without K_2_ZrF_6_ addition. It is also found that all the coatings exhibit a higher and more stable infrared emissivity value after the wavelength of 8 μm. Therefore, the average infrared emissivity of the samples was calculated by the integration method in the wavelength of 8–20 μm and then the ratio was calculated and compared to the blackbody in the same waveband; the result is shown in Figure 7. It can be seen that the average infrared emissivity curves of the coatings present the tendency of increasing firstly and then decreasing slightly. When the concentration of K_2_ZrF_6_ is 6 g/L, the infrared emissivity reaches the maximum and the average infrared emissivity value is 0.89.

The infrared emissivity value of MAO coatings can be affected by a series of factors, such as thickness, surface roughness, chemical composition and structure [33]. Ingeneral, the infrared emissivity increases with the increases of thickness and surface roughness, but the test results did not find a similar regularity. The main reason for this phenomenon is that the effect of chemical composition and structure is much greater than that of thickness and roughness. Therefore, the improvement of infrared emissivity is dependent on the addition of K_2_ZrF_6_ in the electrolyte. In terms of chemical composition, K_2_ZrF_6_ existed as the form of ZrO_2_ in the MAO coatings according to the XPS results. As a king of transition metal oxide, ZrO_2_ itself has high infrared emissivity which can increase the infrared emissivity of the coatings directly. For the internal crystal structure, the lattice defects caused by the Zr^4+^ doping effect is an effective way to promote the lattice vibration, which results in the improvement of the infrared emissivity in the long wavebands. Furthermore, most groups existed in an amorphous state and the disorder of this structure leads to the formation of the local energy levels at amorphous region. The electrons could achieve the transition easily in these local energy levels, resulting in the increase of the infrared emissivity in the short wavebands [34]. When the K_2_ZrF_6_ concentration is 9 g/L, the slight decrease of the infrared emissivity may be due to the formation of brookite-TiO_2_ and the improvement of the crystallization of the coatings [35].

## 4. Discussion

In short, micro arc oxidation is a complex process, the changes in the composition of the electrolyte and the change of the process parameters will have a significant effect on the reaction. According to the above presence of compound peaks in the coatings, the reaction between the TC4 substrate and electrolyte are interpreted as follows:

Ti + 4OH^−^ − 4e^−^ → TiO_2_ + 2H_2_O (1)

2SiO_3_^2−^ − 4e^−^ → 2SiO_2_ + O_2_↑ (2)

The reactions (1) and (2) are achieved by the oxidation of the TC4 substrate. While the reaction (2) is the main reason to promote the silicate deposition, it should be noted that the SiO_2_ existed in the coatings steadily in the form of amorphous state.

2PO_3_^−^ + 2OH^−^ → P_2_O_7_^4−^ + H_2_O (3)

Corresponding to the reaction (3), the PO^3−^ ions produced by the hydrolysis of (NaPO_3_)_6_ will convert to P_2_O_7_^4−^ at high temperature over 800 °C, and it was easy to combine with Na^+^ icons in the solution to form Na_4_P_2_O_7_ according to Hou’s research [31].

(ZrF_6_)^2−^ + 4OH^−^ → Zr(OH)_4_↓ + 6F^−^ (4)

Zr(OH)_4_ → ZrO_2_ + 2H_2_O (5)

SiO_2_ + ZrO_2_ → ZrSiO_4_ (6)

Because of the existence of K_2_ZrF_6_ and NaOH in the solution, the Zr(OH)_4_ colloid precipitation was formed through the chemical reaction between Zr^4+^ and OH^−^. Then the Zr(OH)_4_ could be dehydrated to form ZrO_2_ during the sintering of the MAO process [36,37]. A series of transformations occurred through the routes of reaction (4) and (5). By considering the ZrSiO_4_ which identified in the XPS spectra, the reaction (6) deduces the formation of Zr-Si-O species in the electrolyte under thehigh temperature and high pressure [38,39].

The prepared coatings can be applied to the thermal protection system (TPS) [40], which prevents the heat transferring inward and thus protects the electron apparatus. It is especially applicable for radiating the high friction heat between the aircraft surface and atmosphere and increasing the lifetime of the space vehicles material. However, the MAO coatings maybe cracked and dropped during the extreme environment of high temperature and high pressure. The property of the coatings is affected by many factorsin the process of MAO, how to keep or improve the stability and bond strength of the coatings has become a problem to be researched in future applications.

## 5. Conclusions

In this study, MAO coatings were successfully formed on the TC4 alloy in Na_2_SiO_3_-(NaPO_3_)_6_ based solution with various K_2_ZrF_6_ concentrations. The main results of the present survey are as follows:

1. With the increase of K_2_ZrF_6_ concentration, the surface morphologies of the coatings were changed to dense network structure and the number of discharge holes was significantly increased. The decrease ofthe thickness and the surface roughness also indicated that K_2_ZrF_6_ inhibited the deposition of silicate on the coatings surface.
2. From the XRD analysis, the main phases in the coatings were asanatase-TiO_2_, rutile-TiO_2_ together with Ti phases. The absence of any Si-based, P-based and Zr-based species in the XRD peaks identification and the high-resolution spectra of XPS further confirmed that the elements Si, P and Zr existed in the form of amorphous phase.
3. The infrared emissivity was drastically improved when the K_2_ZrF_6_ was added in the electrolyte. In addition, its highest value was found for the coating with the K_2_ZrF_6_ concentration of 6 g/L and the average could reach 0.89 at wavelength of 8–20 μm. It is considered that the doping of Zr^4+^ and the formation of amorphous ZrO_2_ enhanced the infrared emissivity of the coatings.

## Figures and Tables

**Table 1:** Element content of MAO coatings prepared under different K_2_ZrF_6_ concentrations.

K_2_ZrF_6_ Concetration (g/L)	Element Content of MAO Coatings (at%)							
	O	Na	Si	P	Ti	K	F	Zr
0	71.59	1.41	19.29	2.31	5.39	-	-	-
3	71.97	0.82	18.62	2.54	5.58	0.12	0.10	0.25
6	72.94	0.30	12.15	4.44	7.19	0.10	0.34	2.52
9	72.44	0.30	10.81	4.71	6.60	0.10	1.17	3.87
